# Concomitant Intra-Aortic Balloon Pumping Significantly Reduces Left Ventricular Pressure during Central Veno-Arterial Extracorporeal Membrane Oxygenation—Results from a Large Animal Model

**DOI:** 10.3390/life12111859

**Published:** 2022-11-12

**Authors:** Ilija Djordjevic, Oliver Liakopoulos, Mara Elskamp, Johanna Maier-Trauth, Stephen Gerfer, Thomas Mühlbauer, Ingo Slottosch, Elmar Kuhn, Anton Sabashnikov, Pia Rademann, Alexandra Maul, Adnana Paunel-Görgülü, Thorsten Wahlers, Antje Christin Deppe

**Affiliations:** 1 Department of Cardiothoracic Surgery, University Hospital Cologne, 50937 Cologne, Germany; 2 Department of Cardiac Surgery, Kerckhoff-Clinic Bad Nauheim, Campus Kerckhoff, University of Giessen, 61231 Bad Nauheim, Germany; 3 Division of Thoracic and Cardiovascular Surgery, HELIOS Klinikum Siegburg, 53721 Siegburg, Germany; 4 Department of Cardiothoracic Surgery, Otto-von-Guericke University Magdeburg, 39120 Magdeburg, Germany; 5 Experimental Medicine, Faculty of Medicine and University Hospital of Cologne, University of Cologne, 51109 Cologne, Germany

**Keywords:** ECMO, IABP, cardiogenic shock, left ventricular pressure

## Abstract

(1) Introduction: Simultaneous ECMO and IABP therapy is frequently used. Haemodynamic changes responsible for the success of the concomitant mechanical circulatory support system approach are rarely investigated. In a large-animal model, we analysed haemodynamic parameters before and during ECMO therapy, comparing central and peripheral ECMO circulation with and without simultaneous IABP support. (2) Methods: Thirty-three female pigs were divided into five groups: (1) SHAM, (2) (peripheral)ECMO(–)IABP, (3) (p)ECMO(+)IABP, (4) (central)ECMO(–)IABP, and (5) (c)ECMO(+)IABP. Pigs were cannulated in accordance with the group and supported with ECMO (±IABP) for 10 h. Systemic haemodynamics, cardiac index (CI), and coronary and carotid artery blood flow were determined before, directly after, and at five and ten hours on extracorporeal support. Systemic inflammation (IL-6; IL-10; TNFα; IFNγ), immune response (NETs; cf-DNA), and endothelial injury (ET-1) were also measured. (3) Results: IABP support during antegrade ECMO circulation led to a significant reduction of left ventricular pressure in comparison to retrograde flow in (p)ECMO(–)IABP and (p)ECMO(+)IABP. Blood flow in the left anterior coronary and carotid artery was not affected by extracorporeal circulation. (4) Conclusions: Concomitant central ECMO and IABP therapy leads to significant reduction of intracavitary cardiac pressure, reduces cardiac work, and might therefore contribute to improved recovery in ECMO patients.

## 1. Introduction

Therapy-refractory cardiogenic shock (CS) is associated with high mortality [1]. However, long-term follow-up data sets for survivor of extracorporeal circulation are promising; therefore, short-term survival should be targeted by therapeutic escalation with mechanical circulatory support systems (MCS) in appropriate patients [2]. In general, therapeutic concepts pursue maintaining balanced haemodynamics while supporting affected ventricular function [3]. Several strategies of simultaneously used MCS have been established to achieve best support and goal-directed therapy [4,5]. Despite the current controversial opinion of its doubtful benefit in cardiogenic shock, intra-aortic balloon pumping (IABP) is associated with favourable results in cardiac surgery patients [6]. In particular, concomitant IABP to extracorporeal membrane oxygenation (ECMO) therapy is widely provided in clinical practice. However, incongruent results are available [7,8,9]. Restitution of pulsatility and indirect left ventricular unloading, next to already known beneficial reduction in afterload and increase in coronary perfusion, are major advantages discussed for simultaneous ECMO and IABP therapy [10,11,12]. Brechot and colleagues demonstrated in 259 patients that IABP to ECMO support protects against hydrostatic pulmonary oedema [13]. In this context, IABP utilisation in ECMO circulation is discussed to be a remaining indication in therapy-refractory CS due to its clinically described haemodynamic effects [14]. However, severity and complexity of CS itself might equalise these haemodynamic effects, and therefore short-term outcomes and survival rates might be unaffected. In this regard, few experimental studies are available addressing haemodynamic and cardiocirculatory changes in comparison of single ECMO and ECMO + IABP therapy. Belohlavek and colleagues described in a pig model that in addition to femoro-femoral ECMO circulation IABP support worsens coronary blood flow while increasing coronary perfusion pressure [15]. Next to the option to implement ECMO therapy peripherally via femoral vessels or central via atrio-aortal cannulation, the influence of antegrade (central cannulation) or retrograde (in peripheral cannulation) flow on haemodynamic and organ perfusion during central (antegrade) or peripheral (retrograde) ECMO support is of clinical interest [16]. Schroeter et al. demonstrated in an experimental protocol that antegrade ECMO perfusion with simultaneous IABP is useful, while concomitant IABP impairs perfusion of the coronary arteries in retrograde ECMO circulation [17]. 

Besides haemodynamic effects, ECMO circulation is known to induce and promote a proinflammatory cellular response due to the contact of blood with the foreign surface of the ECMO equipment [18]. The pathophysiologic pathway and amount of proinflammatory protein produced with a single ECMO or concomitant MCS is still a topic of scientific discussion [19]. 

Therefore, we aimed to analyse in a large-animal model the haemodynamic changes of ECMO compared to ECMO and IABP therapy with the establishment of central and peripheral ECMO circulation. The main question was whether concomitant IABP to ECMO support improves haemodynamics. Moreover, inflammatory parameters were investigated to describe the additional impact of concomitant MCS on inflammatory processes.

## 2. Materials and Methods

The responsible ethical committee for animal care of the University of Cologne (LANUV Northrhine-Westphalia, Recklinghausen, Germany) approved the study protocol, and animals were treated in compliance with the Directive 2010/63/EU of the European Parliament. Funding was supported by the Koeln Fortune Programm (343/2015), Faculty of Medicine, University of Cologne. All relevant procedures (experimental protocol and subsequent analysis) were performed between May 2017 and December 2021 at the institution of the Experimental Medicine of the Faculty of Medicine and University Hospital Cologne and the laboratories of the Department of cardiothoracic surgery of the University Hospital Cologne. 

### 2.1. Experimental Protocol 

#### 2.1.1. Groups

**Figure 1:**
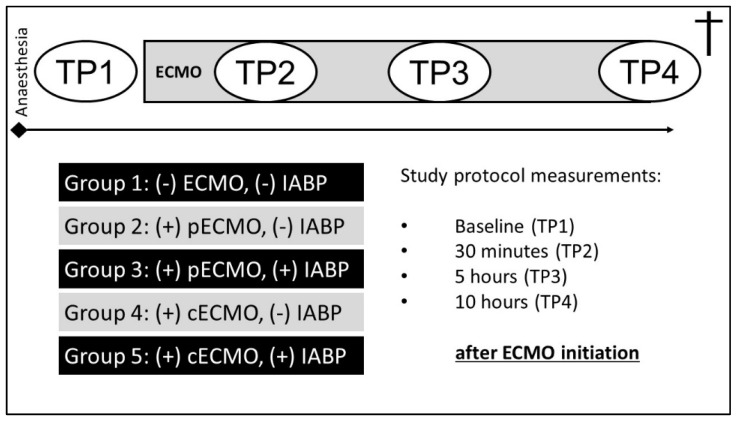
Graphical illustration of the study protocol and investigated groups (TP, time point).

Thirty-three female pigs (Deutsche Landrasse Pietrain, body weight 60.3 ± 4 kg, *n* = 33) were divided into four treatment groups and one control group (SHAM) (Figure 1). The SHAM group did not receive any cardiac assistance [(–)ECMO(–)IABP]. The second group received a peripheral ECMO [(p)ECMO(–)IABP]. In addition to peripheral ECMO treatment, the third group [(p)ECMO(+)IABP] was supported with an IABP. The other groups were treated with a central ECMO [(c)ECMO(–)IABP], and in group five, additionally with an IABP [(c)ECMO(+)IABP].

#### 2.1.2. Instrumentation and Operative Technique

**Figure 2:**
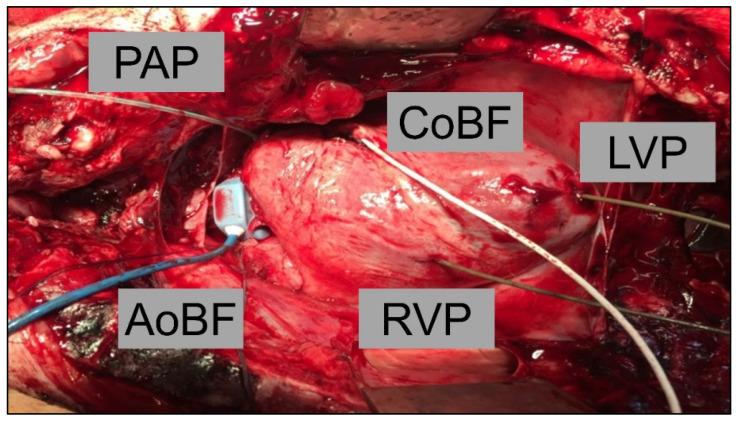
Intraoperative situs with view of catheters and flow probes placed (PAP, pulmonary artery pressure; CoBF, coronoary blood flow; LVP, left ventricular pressure; AoBF, aortic blood flow; RVP, right ventricular pressure).

Animals were premedicated with combinatory intramuscular application of xylazin (2 mg/kg) and zoletil (10 mg/kg). Additionally, pigs received atropin (0.02 mg/kg i.m.). After initiation of sedation with propofol (2 mg/kg i.v.), the animals were endotracheal intubated. Analgo-sedation was maintained with fentanyl (0.012–0.025 mg/kg), midazolam (0.96–1.2 mg/kg), and propofol (4–6 mg/kg) as continuous infusion. Ventilation of pigs was performed by using a volume-controlled ventilator (Fabius GS, Dräger, Lübeck, Germany) with the aim of keeping arterial blood gas parameters in the physiologic range (pO_2_ > 100 mmHg). Achievement of full anaesthesia was continuously examined by the interdigital claw reflex. After sterile washing and covering, a urinary catheter, a central venous catheter (8.5 Fr, ZVK Arrow International, Reading, PA, USA) through the right jugular vein, and an arterial catheter (20 G Leadercath, Vygon, Aachen, Germany) through the right carotid artery were placed surgically. Central venous pressure was kept constant by continuous saline infusion (5–10 mL/kg/h). Mean arterial pressure was adjusted to achieve physiological organ perfusion (>60 mmHg). Morevoer, the contralateral carotid artery was used for measurement of cerebral blood flow with transit time flow probes (Transonic Systems Inc., New York, NY, USA). Afterwards, sternotomy was then performed, and the heart was exposed, wherein a pulmonary catheter (20 G Leadercath, Vygon, Aachen, Deutschland) and 5 Fr pressure transducer-tip catheters (Model SPC-350S, Millar Instruments Inc., Houston, TX, USA) were placed into the pulmonary artery and the left and right ventricles (Figure 2). Cardiac output (Aortic Blood Flow = AoBF) and coronary blood flow (CoBF) were recorded by transit time flow probes (Transonic Systems Inc., New York, NY, USA), which were placed at the ascending aorta and the left anterior descending artery, respectively (Figure 2). 

#### 2.1.3. MCS Implantation

**Figure 3:**
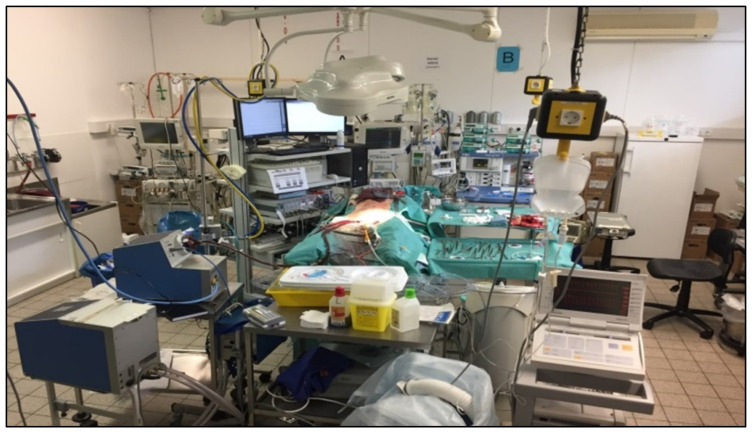
Presentation of the operating room during an ongoing experimental protocol.

The right femoral artery and vein were exposed after systemic anticoagulation (heparin 300 IU/kg), and in the case of peripheral ECMO treatment were cannulated in a wire-guided manner. For central cannulation, the arterial cannula was directly placed into the ascending aorta. A venous cannula was placed in the right atrium in both scenarios. ECMO circulation was performed for 10 h with a flow index of 50 mL/kg/min/m². ECMO equipment consisted of a console (Bio-Medicus 540, Medtronic, Dublin, Ireland), a centrifugal pump (BPX-80 Bio-Pump, Medtronic, Dublin, Ireland), a membrane oxygenator (CAPIOX^®^ FX, Terumo, Shibuya, Japan), a heat exchanger (Bio-Cal 370, Medtronic, Dublin, Ireland), an extracorporeal circuit (Tubing set, Terumo, Shibuya, Japan), and arterial/venous cannulas (Biomedicus 17 Fr art./21 Fr ven., Medtronic, Dublin, Ireland). IABP (97e, Datascope Corp., Fairfield, NJ, USA) were implanted in the aortic arch and placed distally. The thorax was left open until the end of the experiment (Figure 3). After the initiation of extracorporeal circulation, animals were examined for 10 h. Euthanasia was commenced with the application of pentobarbital (80 mg/kg).

#### 2.1.4. Haemodynamic Measurements and Data Analysis

Measurements were performed before ECMO circulation (baseline) and at 30 min, 5 h, and 10 h after ECMO initiation. Heart rate (HR), mean arterial pressure, central and pulmonary venous pressure, the maximal first derivate of left ventricular pressure (LVdp/dt_max_), cardiac index (CI), and blood flow of the carotid and left anterior descending artery were recorded. With a 16-channel haemodynamic set-up, all data were digitised at a rate of 500 Hz and were subsequently analysed (Hugo Sachs Elektronik-Harvard Apparatus GmbH, March-Hugstetten, Germany). 

### 2.2. Biochemical Analysis 

#### 2.2.1. Quantification of Systemically Circulating Cytokines and Endothelin-1

Arterial blood samples were withdrawn before and 10 h after ECMO initiation for measurement of parameters of systemic inflammation (IL-6; IL-10; TNFα; IFNγ) and endothelial injury (endothelin-1, ET-1). Plasma was separated after centrifugation for 10 min at 2000 rpm (4 °C), and samples were stored at –80 °C until assay preparation. Enzyme-linked immunosorbent assays were performed in accordance with manufacturers’ recommendations (ET-1, R&D Systems Inc., Minneapolis, MN, USA; MPO, USCN Life Science Inc., Wuhan, China). A pig Multiplex Immunoassay (ProcartaPlex 4 Plex, Termo Fisher, Waltham, MA, USA) was used to quantify cytokines in plasma samples. All samples were analysed using a Luminex 200 System (Termo Fisher).

#### 2.2.2. Quantification of cfDNA and NETs

Neutrophil extracellular traps (NETs) are an immune response in innate immunity and composed of neutrophil-derived circulating free DNA (cf-DNA). Plasma levels of cfDNA were quantified by Quant-iT Pico Green dsDNA assay by following the manufacturer’s instructions (Invitrogen GmbH, Darmstadt, Germany), as recently reported [20].

#### 2.2.3. Measurement of Reactive Oxygens Species (ROS) in Plasma

ROS levels were measured as described by Wacker and colleagues [21].

### 2.3. Statistical Analysis 

Data are presented as mean ± SD. A two-way analysis of variance (ANOVA) with Fisher’s LSD post hoc test was utilised to analyse intra- and intergroup differences of repeated measurements. Intergroup distinctions for non-repeated measurements were compared by paired or unpaired t-test (SigmaPlot, Systat Software Inc., Erkrath, Germany). *p*-values < 0.05 were considered statistically significant. All experiments and subsequent data analyses were accomplished in a blinded manner for group assignment of animals.

## 3. Results

### 3.1. Haemodynamics and Blood Flow Data

Haemodynamic data are summarised in Table 1. Targeted parameters (CI, pO_2_, MAP) did not differ between the groups. Lower MAP values over experimental time were not taken into account, since they were in the physiological target range after 10 h. HR and CVP did not differ between groups. Haemoglobin values fell significantly off in all groups during the experiment. Decrease was significantly the most in the central ECMO groups when compared to the SHAM (*p* < 0.01). Blood flow in the left anterior coronary and carotid artery did not differ among groups before and after 10 h of extracorporeal circulation.

**Figure 4:**
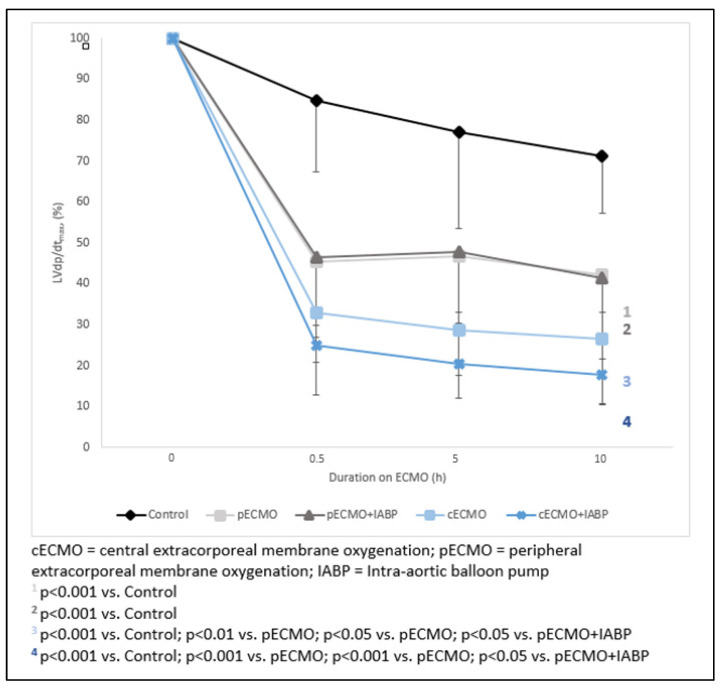
Percentage change of contractility index LVdp/dt (max) before and over 10 h of ECMO treatment.

A significant reduction of RV and LV systolic pressure in comparison to baseline was seen under ECMO therapy in all groups (*p* < 0.001). RVP was significantly lower in all groups when compared to the SHAM. Left ventricular pressure was significantly lower in both central but not in peripheral ECMO groups compared to the SHAM. Concomitant IABP support in (c)ECMO significantly decreased LVP_sys_ compared to peripheral ECMO. Consecutively, a reduction of LVdp/dt_max_ was seen in all ECMO groups. The (p)ECMO(+)IABP group showed no significant difference to the SHAM with regard to LVdp/dt_max_. The percentage change of contractility index is presented in Figure 4. The (c)ECMO(+)IABP group showed the highest change compared to the SHAM. However, additional IABP did not lead to a significant difference in comparison of both central ECMO groups.

### 3.2. Quantification of Plasma Levels of Circulating Cytokines, ET-1, NETs, and ROS

**Figure 5:**
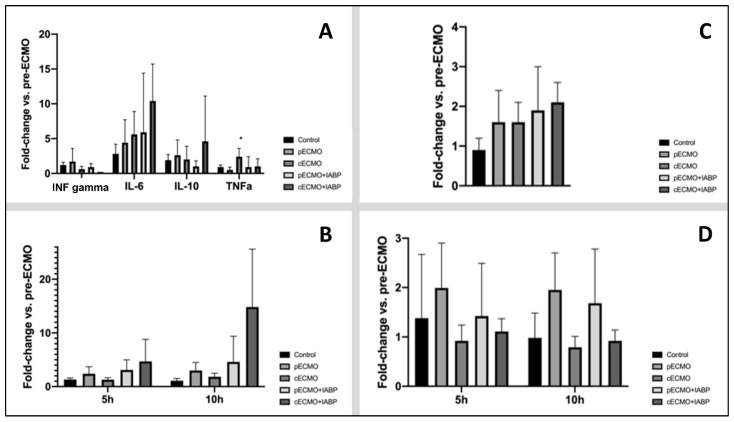
Fold change in systemic release of inflammatory parameters of all groups after 10 h of ECMO treatment compared to pre-ECMO values (mean ± SD, * *p* < 0.05 compared to (p)ECMO(-)IABP). (**A**) Cytokines. Fold change in systemic release of IFNγ, IL-6, IL-10, and TNF-α of all groups after 10 h of ECMO treatment compared to pre-ECMO values. (**B**) NETs. Fold change in systemic release of NETs (neutrophil extracellular traps) of all groups after 5 h and 10 h of ECMO treatment compared to pre-ECMO values. (**C**) ET-1. Fold change in systemic release of endothelin-1 of all groups after 10 h of ECMO treatment compared to pre-ECMO values. (**D**) ROS. Fold change in systemic release of ROS (reactive oxygen species) of all groups after 5 h and 10 h of ECMO treatment compared to pre-ECMO values. cECMO, central extracorporeal membrane oxygenation; pECMO, peripheral extracorporeal membrane oxygenation; IABP, intra-aortic balloon pump.

Plasma levels and fold change in comparison to baseline for circulating cytokines, ET-1, NETs, and ROS are presented in Figure 5. Fold change of IL-6, IL-10, and IFN-γ did not differ between groups (Figure 5A). After 10 h of ECMO therapy, fold change for TNF-α was significantly different in the ((c)ECMO(-)IABP group compared to the (p)ECMO(-)IABP) group. ET-1 levels showed no difference in fold change in comparison to all groups (Figure 5C). No significant changes were obvious for NETs and ROS in all groups (Figure 5B,D).

## 4. Discussion

The present study investigated the impact of concomitant IABP support during ECMO circulation and haemodynamic changes in settings with antegrade and retrograde ECMO flow after central and peripheral cannulation in a large-animal model with 33 healthy pigs. The major findings were

(1) Extracorporeal life support decreased right and left ventricular pressure (RVPsys and LVPsys). The antegrade flow of the central ECMO cannulation was associated with a greater reduction of left ventricular pressure and was enhanced by additional IABP support.
(2) Extracorporeal life support decreased the contractility index (LVdp/dt_max_). The antegrade ECMO flow was associated with a better LV unloading. Furthermore, our data suggest that in the central ECMO group, the IABP may have an optimising effect.
(3) IABP support neither affected coronary nor carotidal blood flow during ECMO circulation, independent of antegrade or central ECMO flow.
(4) Utilisation of extracorporeal circulation did not promote inflammatory cascade.

### 4.1. Haemodynamic Effects of Simultaneous IABP Support during ECMO Circulation

To find the best mechanical circulatory support with the least side effects is of highest clinical relevance. Therefore, there is an ongoing discussion as to whether ECMO therapy should be supported by an IABP. Brechot and colleagues comparatively analysed 259 veno-arterial ECMO patients in regard of additional IABP therapy and their impact on pulmonary oedema [13]. They showed in their multivariable analysis that IABP has been independently associated with a lower risk of radiological pulmonary oedema and a trend towards lower mortality. The concept of ventricular unloading with simultaneous IABP has already been described and is frequently used, although IABP support is an indirect method to unload ventricular cavities [11,22]. Our study group demonstrated with an analysis of 172 ECMO patients with post-cardiotomy cardiogenic shock that, independent of ECMO type, additional IABP support might increase weaning rates off ECMO support [23]. In-hospital mortality rates were not affected in these patients regarding additional IABP use. Recently, Nishi et al. published the largest data to date (*n* = 3815) analysing concomitant ECMO and IABP support versus ECMO alone in CS patients [24]. They demonstrated that patients with VA-ECMO+IABP showed a significantly lower in-hospital, 7-day, and 30-day mortality in comparison to those managed with va-ECMO alone. Li and colleagues corroborated these findings with their retrospective analysis on 2125 patients [9]. In addition, Zeng et al. showed with their meta-analysis including 2573 patients that ECMO combined with IABP can improve in-hospital survival more effectively than ECMO alone in patients with cardiogenic shock [25]. Madershahian et al. showed in post-cardiotomy cardiogenic shock patients that pulsatility induced by IABP improved diastolic filling index and mean coronary bypass graft flows by reducing coronary vascular resistance during peripheral ECMO [26,27]. In contrast to the already proven increase in arterial blood flow (bypass graft) [26,27], our data did not show an increase in coronary or carotid blood flow. Regarding blood flow and arterial pressure, Belohlavek and colleagues investigated 11 pigs in a cardiac arrest model (15). The authors measured arterial pressure and blood flow with intravascular catheter fluoroscopy during central and peripheral ECMO circulation with simultaneous IABP support. In contrast to our data, they showed a significant worsening of coronary blood flow with central ECMO and IABP. Moreover, the experiment demonstrated an increase in coronary perfusion pressure during peripheral ECMO. The authors speculated that the repetitive aortic occlusion, induced by the balloon, may diminish blood flow available for the aortic root more than for the aortic arch. However, the findings by Belohlavek et al. were in accordance with the results of Sauren and colleagues. The study group of Sauren investigated the effect of simultaneous ECMO and IABP support in seven sheep and showed that in the case of low cardiac output and insufficient extracorporeal flow, concomitant IABP to peripheral ECMO circulation may be contraindicated due to proven worsening of haemodynamic parameters [28]. Next to this, the authors showed that independent of central or peripheral ECMO cannulation settings, adjunctive IABP improved the myocardial oxygen supply–demand balance. Schroeter and colleagues provided an experimental study investigating the impact of additional IABP during peripheral and central ECMO circulation on mean coronary arterial pressure (left anterior descending artery) [17]. The study protocol was performed repetitively for 2 min in several settings (100% ECMO flow vs. 50% ECMO flow). The study group concluded that additional IABP support is only useful in antegrade ECMO circulation. Peripheral ECMO canulation leads to a significant decrease in mean coronary arterial pressure with higher myocardial oxygen demand. Moreover, retrograde ECMO circulation without IABP leads to significantly increased pressure in the coronary arteries in comparison to antegrade ECMO circulation [17]. The mentioned experimental setting described short-term changes at the beginning of circulatory support. This might be the reason that our findings in contrast show no differences in coronary or carotid blood blow after 30 min or 10 h. However, our data corroborate the decrease in ventricular and coronary pressure during antegrade ECMO flow. We were able to prove that simultaneous central ECMO and IABP therapy significantly reduced LV pressure in comparison to peripheral ECMO (+/− IABP). Furthermore, reduction in LV pressure was associated with highest reduction of contractility index. In summary, simultaneous use of the IABP with antegrade ECMO flow results in reduction of afterload and left ventricular unloading. In this regard, future studies should focus on this important clinical point that was historically underrepresented in therapeutic measures. Meanwhile, left ventricular unloading and decompression became key points in therapeutic strategies regarding therapy-refractory cardiogenic shock. Therefore, an investigation of the best option to address left ventricular unloading is essential.

### 4.2. Inflammatory Processes Induced by ECMO

The initiation of ECMO is associated with an immediate and complex inflammatory response, in accordance with the systemic inflammatory response syndrome (SIRS) [29]. The inflammatory response is mainly caused by the exposure of blood to the extracorporeal circulation. Both systemic and cellular factors initiate and propagate the SIRS-like cascade. Production of various pro-inflammatory and anti-inflammatory cytokines is one potential reaction [30,31]. Evidence to support a single specific cytokine in this process is minimal. Additionally, whether the amount of MCS systems has an impact on inflammatory response has not been answered. The attempt to downsize cardiopulmonary bypass systems with beneficial effects on systemic inflammation allows for the conclusion that IABP used in addition to ECMO might trigger more inflammatory response. Therefore, our protocol focused on several cytokines. As mentioned, no differences between groups were seen. Additional IABP seems not to impact cytokine production within the first ten hours of extracorporeal circulation. However, TNF-α showed a significant increase after 10 h of extracorporeal circulation in the central ECMO group in comparison to the peripheral ECMO group. Next to potential pathways activated by TNF-α (activator of neutrophils, induction of expression of adhesion molecules), our finding seems unspecific and unclear for evaluation in the setting of our study protocol. Moreover, NETs and cf-DNA were comparable through the groups. Our analysis revealed no difference in ET-1 and ROS between groups.

### 4.3. Limitations

The artificial scenario implanting ECMO and IABP in healthy pigs does not represent a real-life scenario. Moreover, our study protocol in healthy pigs did not aim to produce a pathologic state. Therefore, the investigated data are difficult to transfer onto clinical situations. However, our focus was haemodynamic changes caused by different strategies of extracorporeal life support. Another limitation of the study protocol was the restriction of red blood cell transfusion. To avoid inflammatory trigger by autologous or homologous full-blood transfusion, we had to accept surgically induced anaemia. To reach our targeted parameters (CI, MAP), continuous infusion (crystalloids) was administered. In this regard, haemoglobin values differed significantly depending on the given volume. Thirdly, ten hours of ECMO treatment is too short to show differences in inflammatory response between groups. However, without the possibility of transfusion, extension of the experiment was not feasible. For the same reason, adverse effects of extracorporeal circulation were not addressed. Fourth, due to the concept of final experiments (planned euthanasia), no statement on haemodynamic statement during weaning from ECMO or IABP is feasible. Moreover, follow-up and diagnostics of ventricular function and general haemodynamic state were not possible to assess. Fifth, due to the fact that the thorax was left open, changes in temperature were inevitably artificially promoted and might influence water balance with an impact on the haemodynamic state. In this regard, all results should be valued with caution, and transfer of these results into a human clinical scenario might be difficult.

## 5. Conclusions

Extracorporeal life support decreases right and left ventricular pressure (RVPsys and LVPsys, respectively). IABP support enhances reduction of left ventricular pressure in central canulated ECMO with antegrade flow. The transformation of our animal experimental data into the clinic is pending and should also include clinical endpoints in the evaluation that could not be examined in the final animal model. Simultaneous use of IABP caused no increase in inflammatory response, but longer ECLS running time is mandatory in order to address this topic. However, for better LV unloading and consecutive potentially better recovery, we recommend central canulation with antegrade ECMO flow.

## Figures and Tables

**Table 1:** Haemodynamic data of treatment groups before ECMO and 10 h after ECMO.

Parameter	Group	Before ECMO	10 h on ECMO
HR (bpm)	(-)ECMO(-)IABP	85.6 ± 5.5	90.6 ± 10.2
	(p)ECMO(-)IABP	92.1 ± 9.8	98.4 ± 22.2
	(c)ECMO(-)IABP	96.5 ± 9.7	86.7 ± 6.7
	(p)ECMO(+)IABP	99.6 ± 12.2	91.0 ± 12.1
	(c)ECMO(+)IABP	97.3 ± 5.9	98.0 ± 11.0
MAP (mmHg)	(-)ECMO(-)IABP	68.4 ± 12.7	61.6 ± 10.4 *
	(p)ECMO(-)IABP	71.0 ± 7.4	65.7 ± 9.9 *
	(c)ECMO(-)IABP	77.8 ± 13.3	57.7 ± 3.1 *
	(p)ECMO(+)IABP	73.1 ± 16.3	57.3 ± 5.4 *
	(c)ECMO(+)IABP	74.9 ± 17.0	63.4 ± 7.5 *
CI (L/min/m^2^)	(-)ECMO(-)IABP	3.0 ± 0.2	3.0 ± 0.3
	(p)ECMO(-)IABP	3.0 ± 0.03	3.3 ± 0.4
	(c)ECMO(-)IABP	3.3 ± 0.3	3.5 ± 0.4
	(p)ECMO(+)IABP	3.1 ± 0.4	3.5 ± 0.2
	(c)ECMO(+)IABP	3.3 ± 0.4	3.7 ± 0.6
Hb (mg/dL)	(-)ECMO(-)IABP	8.9 ± 0.8	8.0 ± 1.0 *
	(p)ECMO(-)IABP	8.9 ± 0.6	7.2 ± 1.0 *
	(c)ECMO(-)IABP	8.3 ± 0.8	6.2 ± 0.6 *^;##^
	(p)ECMO(+)IABP	9.1 ± 0.6	6.9 ± 0.8 *
	(c)ECMO(+)IABP	8.2 ± 0.8	6.0 ± 0.8 *^;##;§^
pO_2_, (mmHg)	(-)ECMO(-)IABP	158 ± 38	138 ± 5
	(p)ECMO(-)IABP	142 ± 33	145 ± 36
	(c)ECMO(-)IABP	147 ± 28	150 ± 27
	(p)ECMO(+)IABP	159 ± 24	138 ± 25
	(c)ECMO(+)IABP	148 ± 37	150 ± 37
CVP (mmHg)	(-)ECMO(-)IABP	11.8 ± 1.6	14.1 ± 0.9
	(p)ECMO(-)IABP	12.1 ± 3.2	10.8 ± 1.4
	(c)ECMO(-)IABP	13.1 ± 1.0	12.9 ± 1.3
	(p)ECMO(+)IABP	10.6 ± 3.1	10.5 ± 3.4
	(c)ECMO(+)IABP	11.7 ± 0.6	10.8 ± 3.1
RVPsys (mmHg)	(-)ECMO(-)IABP	29.9 ± 9.5	29.8 ± 6.1
	(p)ECMO(-)IABP	28.1 ± 5.2	15.0 ± 6.8 *^,##^
	(c)ECMO(-)IABP	25.2 ± 2.9	8.7 ± 5.1 *^;###^
	(p)ECMO(+)IABP	23.2 ± 2.8	18.4 ± 4.5 *^;##^
	(c)ECMO(+)IABP	25.2 ± 2.9	11.5 ± 8.7 *^;###^
LVPsys (mmHg)	(-)ECMO(-)IABP	82.4 ± 9.9	79.5 ± 8.6
	(p)ECMO(-)IABP	81.7 ± 7.6	66.4 ± 19.4 *
	(c)ECMO(-)IABP	76.3 ± 13.0	44.3 ± 8.4 *^;##^
	(p)ECMO(+)IABP	87.3 ± 19.0	55.0 ± 14.4 *
	(c)ECMO(+)IABP	75.5 ± 8.1	33.6 ± 2.7 *^;###;§§;+^
LVdp/dt_max_ (mmHg)	(-)ECMO(-)IABP	1729 ± 368	1195 ± 128
	(p)ECMO(-)IABP	1434 ± 429	582 ± 147 *^;##^
	(c)ECMO(-)IABP	1497 ± 162	378 ± 164 *^;##^
	(p)ECMO(+)IABP	1678 ± 628	604 ± 151 *
	(c)ECMO(+)IABP	1484 ± 56	266 ± 114 *^;###^

* *p* < 0.001 vs. before ECMO, ^##^ *p* < 0.01; ^###^ *p* < 0.001 vs. control, ^§^ *p* < 0.05; ^§§^ *p* < 0.01 vs. pECMO, ^+^ *p* < 0.05 vs. pECMO + IABP. bpm: beats per minute; CI: cardiac index; CVP: central venous pressure; Hb: haemoglobin; HR: heart rate; LVdp/dt_max_: maximal first derivate of left ventricular pressure; LVPsys: left ventricular systolic pressure; MAP: mean arterial pressure; pO_2_: partial pressure of oxygen; RVPsys: right ventricular systolic pressure.

## Data Availability

Data supporting reported results can be provided on request of the corresponding author.
